# The Role of PAM4 in the Management of Pancreatic Cancer: Diagnosis, Radioimmunodetection, and Radioimmunotherapy

**DOI:** 10.1155/2014/268479

**Published:** 2014-04-10

**Authors:** Suxia Han, Guihua Jin, Lijuan Wang, Meng Li, Chenchen He, Xijing Guo, Qing Zhu

**Affiliations:** Department of Medical Oncology, The First Affiliated Hospital of Xi'an Jiaotong University Medical Center, Xi'an, Shannxi 710061, China

## Abstract

PAM4, a new monoclonal antibody (MAb) known as clivatuzumab, is highly reactive with pancreatic cancer and precursor lesions. It is absent from the normal tissues and has limited reactivity with nonpancreatic cancer. The detailed characteristic of the PAM4 epitope is unknown but recent studies have shown that it is dependent on MUC1 glycosylation status. The limited PAM4 expression pattern makes it an attractive candidate for management of pancreatic adenocarcinoma. In addition, PAM4 is a serum biomarker for diagnosis of pancreatic cancer. Several different radiolabeled immunodiagnostic and immunotherapeutic agents of PAM4 have been developed and some are being evaluated in preclinical and/or clinical studies. The review will focus on PAM4 and its potential utility for the diagnosis, radioimmunodetection, and radioimmunotherapy of pancreatic cancer.

## 1. Introduction

PAM4, a new monoclonal antibody (MAb) also known as clivatuzumab, is absent from the normal tissues, as well as breast cancer, liver cancer, prostate cancer, and renal cancer. It is reactive with greater than 80% of pancreatic cancer and has a limited reactivity with ovarian cancer, stomach cancer, colon adenocarcinoma, and lung cancer [1–3]. In addition, PAM4 is also expressed in its precursor lesions, pancreatic intraepithelial neoplasia (PanIN), and intraductal papillary mucinous neoplasia (IPMN) in pancreatic cancer [3].

Pancreatic cancer is one of the deadliest of the solid malignancies with a 5-year survival rate of 3–5% [4, 5]. It is the fourth commonest cause of cancer-related death among men and women in the United States. In 2013, an estimated 45,220 people in the USA were diagnosed with pancreatic cancer, and 38,460 died of the disease [6]. Most cases of pancreatic cancers have advanced stage at time of diagnosis with a median survival of less than 1 year [7]. The dismal prognosis can be partly attributed to the absence of early symptoms, late diagnosis, and the poor response to radio- and chemotherapy. How to establish a methodology to define benign pancreatitis form pancreatic malignancy or metastatic carcinomas remains to be investigated. Although CA19-9 is the most widely investigated and evaluated marker for testing pancreatic cancer diagnosis, the sensitivity and specificity are not optimal. With rapid advances in imaging technology, ultrasound, computerized tomography (CT), magnetic resonance imaging (MRI), positron emission tomography (PET), and PET-CT technologies play an important role in the diagnosis of pancreatic cancer. But it cannot reliably estimate small lesions [8, 9]. Surgical resection has been the only modality curative treatment for pancreatic cancer, but the majority of patients present at a late stage when the disease does not respond to surgical therapy [10]. Radiation and/or chemical therapy have a limited impact on the control of pancreatic cancer, resulting in the rapid regrowth of the tumor [7, 11]. Thus, there is an urgent need to develop new means for early diagnosis and new therapeutic approaches to improve the clinical outcome of the deadly disease. Monoclonal antibody diagnosis and therapy represent a new promising approach. In the review, PAM4 is discussed with a focus on its potential as a serum marker for diagnosis and as a target of both radioimmunodiagnostic and radioimmunotherapeutic agents in pancreatic cancer due to its limited distribution on normal tissues and other solid tumors.

## 2. Characteristics of PAM4 

PAM4, a monoclonal antibody to MUC1, is an lgG1 immunoglobulin produced by immunization of mice with mucin purified from the xenografted RIPI human pancreatic cancer—originally a mucinous, moderately differentiated tumor in the head of the pancreas [2]. MUC1 is a transmembrane glycoprotein associated with cell transformation, invasion, migration, apoptosis, cellular interactions, immune regulation, and drug resistance [12–16]. PAM4 recognizes a unique and novel epitope which is not reactive with the peptide core of mucin and distinct from that of B72.3, CA19-9, DUPAN2, Span1, Nd2, CEA, and Lewis antigens [2, 17]. Recent studies show that PAM4 is reactive with the C-terminal region of the MUC5AC [1]. The PAM4 epitope is a conformationally dependent peptide epitope. Actually the carbohydrate structures are not the part of the PAM4 epitope but are necessary to maintain the correct peptide conformation [2]. Furthermore, the PAM4 epitope was found to be highly sensitive to heat, reduction of disulfide bonds, proteolytic digestion, or deglycosylation. In addition, the epitope was partially sensitive to periodate oxidation or neuraminidase digestion. Although the detailed characteristic of the PAM4 epitope is unknown, it is thought to be dependent on MUC1 glycosylation status in recent studies.

## 3. Reactivity of PAM4 with Pancreatic Cancer and Precursor Lesions 

There is a growing body of evidence that PAM4 is highly reactive with pancreatic cancer. In the original study by Gold and coworkers, the immunoreactivity of PAM4 with pancreatic cancer was evaluated by immunohistochemistry using frozen section tissues of patients [2]. All but four pancreatic cancers (21/25, 23 primary and 2 metastatic) were immunoreactive with PAM4. PAM4 reactivity showed weak positive staining of 40% (10 of 26) of colorectal cancer, 20% (1 of 5) of gastric cancer, and 6.6% (1 of 15) of lung cancer. Interestingly, staining was restricted to the ductules; minor staining of a few scattered ductule cells was observed. None of the breast cancer, ovarian cancer, liver cancer, prostate cancer, and renal cancer was stained. However, compared to its weak staining in the goblet cells along the gastrointestinal tract, strong staining was present in pancreatic cancer. This was confirmed in a large study by Gold et al. who evaluated the immunoreactivity with PAM4 in 320 invasive cancer specimens by tissue microarrays [3]. In the study, PAM4 expression was present in 48 of 55 (87%) pancreatic cancers, 6 of 40 (15%) stomach cancers, 7 of 76 (9%) colon adenocarcinomas, 4 of 24 (17%) ovarian cancers, 4 of 40 (10%) lung cancers, 0 of 50 (0%) breast cancers, and 0 of 35 (0%) hepatocellular cancers.

Invasive pancreatic cancer can arise from three different noninvasive precursor lesions, including pancreatic intraepithelial neoplasias (PanIN), intraductal papillary mucinous neoplasms (IPMN), and mucinous cystic neoplasms (MCNs) [18, 19]. A hypothesis that has been proposed is that PAM4 may be present during precursor lesions transition to invasive pancreatic adenocarcinoma. Gold et al. assessed the expression of the PAM4-reactive MUC1 by immunohistology in a study cohort of 55 invasive adenocarcinomas, 63 pancreatic intraepithelial neoplasias (PanIN), 36 intraductal papillary mucinous neoplasms (IPMN), and 11 normal pancreases [3]. PAM4-reactive MUC1 was absent from normal pancreas, but it was identified in 87% of invasive cancers with no striking correlation with the clinical stage of disease. There was a trend for those tumors that were better differentiated to show the higher expression of the PAM4-reactive MUC1. Most importantly, PAM4 is abundantly present in the earliest stages of pancreatic adenocarcinoma and its expression remains high in all stages of PanIN patients. For example, PAM4 labeled 94% (44 of 47) of the PanIN patients tissues in the earliest stage (stages 1A and 1B), 91% (10 of 11) of stage II, 40% (2 of 5) of stage III, and 86% (31 of 36) of IPMN. On the basis of these studies, it is concluded that PAM4 is highly restricted to pancreatic adenocarcinoma and its precursor lesions, but it is also expressed to a lesser degree in other solid tumors.

## 4. Detection of PAM4 in Sera of Pancreatic Cancer Patients

The current most commonly available serum biomarker for pancreatic cancer is CA19-9. It is not useful in detecting early cancers and defining pancreatic cancer from pancreatitis and other benign lesions due to its poor specificity and sensitivity. Thus, the novel serum makers are required for the diagnosis of pancreatic cancer.

PAM4 can be shed from tumors and detected in serum. In recent years, the role of serum PAM4 in the diagnosis of pancreatic cancer has been evaluated. Superior values with 82% sensitivity and 95% specificity were observed by Gold et al. for PAM4 in a well-defined study group of 68 carcinomas, 29 chronic pancreatitides, and 19 healthy volunteers [20]. Apart from overall diagnostic performance of PAM4 in the study, stage-dependent evaluation showed increasing sensitivities at advanced tumor stages with a sensitivity of 62% at stage I (*n* = 21), 86% at stage II (*n* = 14), and 91% at advanced stages 3 and 4 (*n* = 33). Another article by Gold et al. examined the presence of PAM4-reactive MUC1 as a serum marker for pancreatic cancer with a sensitivity of 77% and a specificity of 95% [21]. A total of 283 subjects were evaluated, including 53 pancreatic cancer patients, 87 pancreatitis patients, 100 other cancer patients, and 43 healthy volunteers. As with the PAM4 immunoassay, none of the healthy specimens and only four of 87 pancreatitis patients (5%) were positive above a cut off of 10.2 units/mL. However, of the 87 pancreatitis samples, the positive rate of CA19-9 was 37%. A direct pairwise comparison of PAM4 and CA19-9 immunoassays for discrimination of pancreatic cancer and pancreatitis resulted in a significant difference, with the PAM4 immunoassay demonstrating superior sensitivity and specificity.

Approximately seven years later, Gold tested the PAM4 in a large study group of 298 pancreatic ductal adenocarcinomas (PDAC), 99 other cancers, 120 benign pancreases, and 79 healthy controls reaching 76% sensitivity and 96% specificity [22]. The study was performed blindedly. The specificity was significantly greater for the PAM4 assays than CA19-9 assays, particularly with regard to chronic pancreatitis (86% and 68%, resp.). Besides good overall high diagnostic performance, the detection rate for patients with respect to stage I disease was 64% and was considerably higher for patients with advanced disease (85%). PAM4 antigen levels were significantly higher in patients with PDAC than in other patient groups. At the same time, they evaluated the combination of PAM4 and CA19-9 in serial testing and obtained an improved sensitivity (84%) for the overall detection of PDAC without a significant loss of specificity (82%) compared with either assay alone in 474 specimens. Some reported that both PAM4 and CA19-9 were present simultaneously in the same serum, but others reported that they were independent of each other. For the most part, sera level from patients with pancreatic cancers arising from other tissues of origin did not have detectable levels of the PAM4 antigen.

From these studies, serum PAM4 was reactive with a higher percentage of pancreatic cancer and gave a greater overall intensity of reaction at equivalent concentrations compared to serum CA19-9. It also appeared that PAM4 showed a superior sensitivity and specificity for discrimination of pancreatic cancer from pancreatitis than did CA19-9. Combining PAM4 and CA19-9 can lead to an improvement in diagnostic accuracy for discriminating pancreatic cancer from pancreatitis and other solid tumors. In the near future, their role in clinical practice will be very important to diagnosis. On the other hand, it is obvious that a serum-based biomarker would provide a clinically more valuable and cost-effective tool for the early detection and diagnosis of pancreatic cancer.

## 5. PAM4 for Radioimmunodetection of Pancreatic Cancer

Modern imaging modalities, like ultrasound, CT, MRI, PET, and PET-CT, provide essential information for detection, diagnosis, and management of pancreatic cancer [8, 9]. However, there are many limitations with respect to the detection of small lesions, as well as for discriminating pancreatic cancer and precursor lesions from pancreatitis. Detection of small, early stage pancreatic adenocarcinoma in the asymptomatic patient is crucial for the increased survival rates. Monoclonal antibody (MAb) based imaging holds the potential to impact these problems. Taking into account the high specificity of PAM4 in pancreatic cancer, several lines of work support the use of PAM4-based radioimmunotargeting agents for pancreatic cancer imaging. There are several radiolabeled PAM4 agents that have been developed and some are being evaluated in preclinical and/or clinical studies, such as ^131^I-PAM4, ^111^In-PAM4, ^99m^Tc-PAM4, and ^90^Y-PAM4.

More than a decade ago, Gold and colleagues used postadministration imaging of ^131^I-labeled murine PAM4 (^131^I-mPAM4) to assess tumor targeting in the four different human pancreatic cancer models (AsPc1, BXPC3, Hs766T, and CaPan1) that represent the range of expected differentiation of this tumor type [23]. After intravenous administration of ^131^I-mPAM4 there was preferential localization of radioactivity in each tumor line as assessed by the tumor : nontumor ratios and tumor : blood ratios. Tumor/nontumor ratios for PAM4 were always greater than for nonspecific, isotype-matched Ag8. At the same time, the blood activity of ^131^I-mPAM4 was significantly lower than Ag8 over the period of observation. Peak levels of radioactivity in tumor were identified at day 1 after injection. And the specific tumor concentration of PAM4 increased levels of PAM4 protein (from 10 *μ*g to 100 *μ*g). There was no evidence of PAM4 targeting to nontumor tissues except for splenic uptake in CaPan1 tumor, which may be due to that PAM4 released from the tumor became entrapped in the spleen or that circulating antigen-antibody complexes were deposited in the spleen. Other studies also showed similar results [24, 25]. In these studies, radiolabeled PAM4 showed specific localization of the primary orthotopic and metastatic tumors without significant accumulation in noncancer sites. Of further note, microautoradiography was performed on 5 *μ*m sections through the tumor.

In initial clinical trials by Mariani et al., ^131^I-mPAM4 was injected into five patients with suspected pancreatic cancer (postoperation pathology confirmed pancreatic cancer in four of the five patients, whereas the fifth patient was diagnosed with chronic pancreatitis) [17]. They found a quite satisfactory pattern of distribution of the ^131^I-mPAM4 with good radioactivity accumulation in primary pancreatic cancer and metastatic pancreatic cancer. Furthermore, they found no severe adverse clinical reactions and no abnormal results of blood chemistry tests, whereas low nonspecific radioactivity accumulation in the liver, spleen, and bone marrow was due to the blood pool. Immunoscintigraphy showed clear tumor uptake in all four pancreatic cancer patients, but failed to find in the pancreatitis patients. Significant uptake of ^131^I-mPAM4 in the tumor lesions was observed at relatively late times, starting about 72–96 hours after tracer injection. The tumor lesions detected by immunoscintigraphy ranged in size from bulky lesions to liver metastases about 1-2 cm. In another study of five metastatic pancreatic cancer patients who received ^131^I-mPAM4 IgG (*n* = 2) or ^99m^Tc-mPAM4 Fab' (*n* = 3), Gold et al. observed definitive tumor localization in four of five patients; the fifth had no staining with mPAM4 by immunohistology [24]. The overall sensitivity for detection was 66%. Consistent with previous findings, mPAM4 specifically targetd not only can primary tumors but also metastatic lesions in pancreatic cancer patients. These data indicate the favorable tumor-targeting potential of radiolabeled mPAM4 for diagnostic in primary and metastatic pancreatic cancer patients by immunoscintigraphy.

Murine MAbs have a short survival time and induce a human anti-mouse antibody (HAMA) response. Thus humanized PAM4 (hPAM4) and chimeric PAM4 (cPAM4) based on mPAM4 are being evaluated in preclinical and/or clinical studies. The specificity and biodistribution characteristics of ^125^I-labeled cPAM4 (^125^I-cPAM4) and ^111^In-labeled cPAM4 (^111^In-cPAM4) were shown to be similar to that of ^131^I-mPAM4 as described above [24, 26]. In these studies, accumulation of cPAM4 within the CaPan1 tumor-bearing mice was 2.8-fold higher than nonspecific hLL2 (anti-CD22 antibody), with peak levels of cPAM4 occurring on day 4 after injection. Tumor/nontumor ratios for cPAM4 were always greater than for hLL2. Tumor/blood radiation dose ratios were 3.6 and 0.6 for ^90^yttrium-1, 4, 7, 10-tetraazacyclododecane-N, N′, N′′, N′′′-tetra-acetic acid (DOTA) cPAM4 (^90^Y-DOTA-cPAM4) and ^90^Y-DOTA-hLL2, respectively. Gulec et al. investigated the biodistribution of ^111^In-labeled humanized PAM4 (^111^In-hPAM4) in patients suffering from pancreatic cancer in a phase I clinical trial [27–29]. All ^111^In-hPAM4 images revealed a normal antibody biodistribution pattern. Pancreatic cancers were clearly visible at 24 hours after injection and the visibility was progressively prominent on the subsequent images. There was no qualitative difference apparent in the biodistribution of ^111^In-hPAM4 at the two doses (10 mg and 100 mg total hPAM4). Thus, the hPAM4 was suitable for the scintigraphic visualization of patients with pancreatic cancer.

The group of Cardillo developed bsPAM4, a divalent and bispecific F(ab')2 MAb, that was generated from chimeric PAM4 Fab' and murine 734 Fab' fragments and then used in conjunction with 2 peptide haptens, ^111^In-labeled Ac-Phe-Lys (DTPA)-Tyr-Lys (DTPA)-NH2 (^111^In-IMP-156) and ^99m^Tc-labeled Ac-Lys (DTPA)-Tyr-Lys (DTPA)-Lys (thiosemicarbazonyl-glyoxyl-cysteinyl-)-NH2 (^99m^Tc-IMP-192) [30–33]. To confirm the tumor targeting specificity and delivery of bsPAM4 to the tumor, the biodistribution of this bsPAM4 was investigated in CaPan1 tumor-bearing mice using ^125^I-labeled bsPAM4 (^125^I-bsPAM4). They found significantly higher amount of radioactivity in the tumor with ^125^I-bsPAM4 as compared with nontargeting ^131^I-labeled bsRIT antibody and significantly greater tumor : nontumor ratios with ^125^I-bsPAM4 than directly radiolabeled PAM4 F(ab')2 or PAM4 whole IgG. By immunoscintigraphy, tumors could be visualized as early as 0.5 hour after injection. Furthermore, it is demonstrated that both ^111^In-IMP-156 and ^99m^Tc-IMP-192 were suitable to detect pancreatic adenocarcinoma xenograft tumors pretargeted with bsPAM4. Approximately 4 years later, Gold and colleagues developed a novel humanized tri-Fab bispecific antibody. The bispecific antibody, TF10, was divalent form MAb-PAM4 and monovalent for MAb-679 and can react against the histamine-succinyl-glycine hapten [34–36]. Biodistribution studies and nuclear imaging of the radiolabeled TF10 and/or TF10-pretargeted hapten-peptide (IMP-288) were conducted in nude mice bearing CaPan1 human pancreatic cancer xenografts. They found greater tumor : nontumor ratios for TF10-pretargeted ^111^In-IMP-288 as compared with ^111^In-IMP-288 alone and ^111^In-hPAM4 alone and superior images for TF10-pretargeted ^111^In-IMP-288 compared with ^111^In-IMP-288 alone. Tumor uptake of TF10 was >100-fold higher than ^111^In-IMP-288 alone. Moreover, TF10 cleared rapidly from the blood and nontumor tissues, while maintaining high signal strength at the tumor site. Of special note, the majority of these tumors were ≤0.5 cm in diameter. These studies demonstrated the feasibility of the pretargeted bsPAM4 and TF10 for nuclear imaging of human pancreatic cancer xenograft tumors in nude mice.

Thus, PAM4-based radioimmunoimaging is not only used for determining focal, size, range, and location of pancreatic cancer, but also for early detecting of the small lesions which cannot be detected by conventional imaging modalities.

## 6. PAM4 for Radioimmunotherapy of Pancreatic Cancer

Besides the usefulness of PAM4 as a target of radioimmunodiagnostic agents, radiolabeled PAM4 has also been tested for selective treatment of pancreatic cancer. hPAM4 radiolabelled with *β*-emitting radioisotopes, such as yttrium-90 (^90^Y), has been used for the radioimmunotherapy (RAIT) of pancreatic cancer in clinical trials.

Animal studies consistently show that administration of ^131^I-PAM4 to orthotopic transplants of CaPan1 tumors exhibited marked regression of tumors and significantly (*P* < 0.001) extended survival time with few toxic effects as compared with untreated control group [25, 29, 37]. ^131^I-PAM4 provided at least 3-fold longer extended survival time than untreated controls in animals bearing tumors of 0.5 and 1.0 cm^3^ groups and more than 2-fold increase in the very large tumor burden of 2.0 cm^3^. Furthermore, it provided cure for small tumors (≤0.25 cm^3^) and a *∼*60% cure rate for large tumors (1.0 cm^3^). On the other hand, a significant extended survival was observed for the group receiving 2 doses compared to the group receiving only 1 dose in tumor burden of 1.0 cm^3^. ^90^Y may prove the superior option because it has a greater energy emission, shorter half-life, and longer path length of radiation emission than ^131^I [26]. Thus, Cardillo et al. compared the antitumor effects in mice bearing CaPan1 xenograft tumors (~1.0 cm^3^) between the ^90^Y-PAM4 and ^131^I-PAM4. They found that ^90^Y-PAM4 provided significantly greater growth inhibition and longer median survival time than the ^131^I-PAM4 (*P* < 0.035). These studies provide a rationale for initiating phase I clinical study for therapy of pancreatic cancer with PAM4.

Recent studies have looked at the antitumor activity of the radiolabeled PAM4 in combination with gemcitabine chemo/radiosensitization agent [17, 38–40]. CaPan1 xenograft model was treated with gemcitabine alone, low dose ^131^I-cPAM4 or ^90^Y-DOTA-cPAM4 alone, or the two agents in combination. This study showed marked antitumor synergy when gemcitabine was combined with low dose ^131^I-cPAM4 or ^90^Y-DOTA-cPAM4. Increased antitumor activity with prolonged median survival time was doubled for the combined treatment regimen compared with treatment with gemcitabine alone and low dose ^131^I-cPAM4 alone. Furthermore, two cycles of the combined ^90^Y-DOTA-cPAM4 and gemcitabine yielded significant tumor regression and increased median survival compared to only 1 cycle, gemcitabine alone, ^90^Y-DOTA-cPAM4 alone, and those untreated. It is important to note that gemcitabine did not interfere with the biodistribution of radiolabeled antibody. A similar set of studies on treatment with double the dose of ^131^I-cPAM4 with gemcitabine resulted in superior antitumor activity with tumor growth inhibition and 2-fold increased median survival time than treatment with ^131^I-hLL2 (a isotype-matched, humanized LL2 anti CD22 antibody) with gemcitabine.

Given the higher accumulation of radioactivity with bsPAM4 to pancreatic cancer, Gold and colleagues conducted studies to evaluate bsPAM4 as an immunotargeting agent for pancreatic cancer therapy. In recent years, they pursued the efficacy of TF10/^90^ Y-peptide pretargeting in CaPan1 xenografts model [34]. Radiation dose estimates suggested that TF10/^90^ Y-peptide pretargeting would provide a greater antitumor effect than ^90^Y-PAM4. In another study by Karacay and colleagues, TF10-^90^Y-IMP-288 combined with gemcitabine in CaPan1 xenografts model significantly enhanced survival as compared with TF10-^90^Y-IMP-288 alone [41]. Moreover, weekly fractionation of the PT-RAIT (pretargeted radioimmunotherapy) improved the responses as compared with a single treatment. Thus, PAM4-based PT-RAIT with ^90^Y hapten peptide is an effective treatment for pancreatic cancer in animal model, and when combined with dose fractionation of the PT-RAIT, further improvements in therapeutic response were observed. With the current preclinical RAIT-gemcitabine studies, it is important to note that a significant and substantial antitumor effect was observed without evidence of life-threatening toxicity.

In the study by Glazer and colleagues, tumor sizes were measured weekly by exposing Panc-1 and CaPan1 human pancreatic carcinoma xenografts to an RF (radiofrequency) field 36 hours after treatment with PAM4 antibody-conjugated AuNPs (gold nanoparticles) [42]. They found that CaPan1 tumors exposed to RF fields after PAM4-AuNP treatment were significantly smaller and began between weeks 1 and 2. Furthermore, both Panc-1 and CaPan1 tumors treated with PAM4-AuNP followed by RF field exposure were necrotic compared with control tumors treated with RF field exposure or PAM4-AuNP alone. Importantly, this occurred without any evidence of injury to selected normal tissues (liver, spleen, lung, and kidney), changes in animal behavior and habits, or unexplained animal death throughout the course of the experiment.

Two phase I clinical trials of ^90^Y-clivatuzumab tetraxetan (^90^Y-hPAM4) have been completed. These studies were designed to test the safety, maximum tolerated dose (MTD), tumor responses, immunogenicity, and pharmacokinetics of ^90^Y-hPAM4. In one study, 21 patients (17 patients with stage IV and 4 patients with stage III) initially received 3 to 5 mCi of ^111^In-hPAM4, followed 1 week later by a single dose of ^90^Y-hPAM4 with ^90^Y doses of 15 (*n* = 8), 20 (*n* = 9), and 25 mCi/m^2^ (*n* = 4) [27]. However, one patient withdrew before ^90^Y-hPAM4 at the 15.0 mCi/m^2^ dose level after developing gastric outlet obstruction from disease progression. Administration was well tolerated in all patients and the MTD of ^90^Y-hPAM4 was established at 20 mCi/m^2^, with expected dose-limiting myelosuppression. Antitumor activity was assessed by CT-based Response Evaluation Criteria in Solid Tumors (RECIST). Out of the 20 patients treated, 3 patients had transient partial response and 4 patients had stable disease at 4-week evaluations. They achieved 32% to 52% shrinkage of the sum of the longest diameters of their target lesions at 4-week evaluations. The median PFS for all 20 patients was 4.3 weeks. The median overall survival (OS) was 4.3 months, including 6 patients surviving 9.3 to 22.2 months. In particular, the treatment of smaller-sized tumors seems to be attractive as it was demonstrated that median OS are approximately 3.5 times longer in maximum lesions ≤4.5 cm than in the larger lesions. Immunogenicity analysis showed that the majority of the treated patients were negative. Pharmacokinetic analysis showed that the serum of ^111^In-PAM4 was 4.6 ± 0.9 days for initial 15 patients and 3.8 ± 0.5 days for the final 6 patients. CA19-9 velocity stabilized or decreased for 4 weeks after treatment in 5 of 20 patients (25%). In the other study, patients received gemcitabine 200 mg/m^2^ weekly for 4 weeks with ^111^In-PAM4 given in the first week and ^90^Y-hPAM4 given once weekly for the next 3 weeks (cycle 1) [29]. 19 patients receive dose-escalation weekly ^90^Y doses of 6.5 mCi/m^2^, 9.0 mCi/m^2^, 12.0 mCi/m^2^, and 15.0 mCi/m^2^. 19 patients subsequently received weekly doses of 9.0 mCi/m^2^ or 12.0 mCi/m^2^. A total of 38 patients (33 patients with stage IV and 5 patients with stage III) were treated using this schedule and the MTD of ^90^Y-hPAM4 was 12 mCi/m^2^ weekly for 3 weeks for cycle 1, with 9.0 mCi/m^2^ weekly for 3 weeks for subsequent cycles. The dose-limiting toxicity (DLT) included grade 3/4 thrombocytopenia or neutropenia in 28 of 38 patients after cycle 1 and in all retreated patients. ^90^Y-hPAM4 was well tolerated with infusion reaction. Out of the 38 patients treated, 6 patients (all with stage IV disease) had partial response and 16 had stable disease as their best response. The 38 treated patients had a median overall survival (OS) of 7.7 months, including 11.8 months for those who received repeated cycles (46% (6 of 13 patients) ≥ 1 year), with improved efficacy at the higher radioimmunotherapy doses. In only one patient, an elevated titer of anti-hPAM4 antibodies (HAHA) was observed. Pharmacokinetics varied between the first cycle and the second cycle. With respect to CA19-9, 33% of patients had a decrease of >50% and 27% of patients had a decrease of >75% from baseline after the first cycle at all dose levels. The results of this study showed that ^90^Y-hPAM4 is safe and had antitumor activity in pancreatic cancer patients.

## 7. Conclusions and Perspectives

PAM4 is an lgG1 immunoglobulin that has limited reactivity with nonpancreatic cancers and is absent from the normal pancreas. PAM4 is highly reactive with pancreatic adenocarcinoma and its precursor lesions, which makes it a good candidate for pancreatic cancer detection and therapy. In serum analysis, PAM4 has a superior sensitivity and specificity for pancreatic cancer compared to CA19-9. Combining PAM4 and CA19-9 can lead to an improvement in diagnostic accuracy for discriminating pancreatic cancer from pancreatitis and pancreatic cancer from other solid tumors. Taken together, PAM4 not only is a good biomarker for pancreatic cancer diagnosis, but also might be a supplementary marker for pancreatic cancer diagnosis. Thus far, preclinical and clinical trials of radiolabeled PAM4 as a target of both immunodiagnostic and immunotherapeutic agents have shown great potential in the imaging and therapy of pancreatic adenocarcinoma. Overall, PAM4 is a promising new means to explore. In the near future, PAM4 may be implemented into clinical routine for diagnosis, radioimmunodetection, radioimmunotherapy, and management of pancreatic adenocarcinomaand, possibly, may be seen in the field of radioimmunoguided surgery.

## References

[1] Gold DV, Newsome G, Liu D, Goldenberg DM (2013). Mapping PAM4 (clivatuzumab), a monoclonal antibody in clinical trials for early detection and therapy of pancreatic ductal adenocarcinoma, to MUC5AC mucin. *Molecular Cancer*.

[2] Gold DV, Lew K, Maliniak R, Hernandez M, Cardillo T (1994). Characterization of monoclonal antibody PAM4 reactive with a pancreatic cancer mucin. *International Journal of Cancer*.

[3] Gold DV, Karanjawala Z, Modrak DE, Goldenberg DM, Hruban RH (2007). PAM4-reactive MUC1 is a biomarker for early pancreatic adenocarcinoma. *Clinical Cancer Research*.

[4] Raimondi S, Maisonneuve P, Lowenfels AB (2009). Epidemiology of pancreatic cancer: an overview. *Nature Reviews Gastroenterology and Hepatology*.

[5] Siegel R, Naishadham D, Jemal A (2013). Cancer statistics, 2013. *CA: A Cancer Journal for Clinicians*.

[6] Yadav D, Lowenfels AB (2013). The epidemiology of pancreatitis and pancreatic cancer. *Gastroenterology*.

[7] Hidalgo M (2010). Pancreatic cancer. *The New England Journal of Medicine*.

[8] Raman SP, Horton KM, Fishman EK (2012). Multimodality imaging of pancreatic cancer—computed tomography, magnetic resonance imaging, and positron emission tomography. *The Cancer Journal*.

[9] Gaa J, Fingerle AA, Holzapfel K, Rummeny EJ (2009). MRI for malignant pancreatic tumors. *Radiologe*.

[10] Saif MW (2007). Controversies in the adjuvant treatment of pancreatic adenocarcinoma. *Journal of the Pancreas*.

[11] Michl P, Gress TM (2013). Current concepts and novel targets in advanced pancreatic cancer. *Gut*.

[12] Xu X, Bai L, Chen W (2012). MUC1 contributes to BPDE-induced human bronchial epithelial cell transformation through facilitating EGFR activation. *PLoS ONE*.

[13] Cascio S, Farkas AM, Hughey RP, Finn OJ (2013). Altered glycosylation of MUC1 influences its association with CIN85: the role of this novel complex in cancer cell invasion and migration. *Oncotarget*.

[14] Chen Q, Li D, Ren J (2013). MUC1 activates JNK1 and inhibits apoptosis under genotoxic stress. *Biochemical and Biophysical Research Communications*.

[15] Agrawal B, Gendler SJ, Longenecker BM (1998). The biological role of mucins in cellular interactions and immune regulation: prospects for cancer immunotherapy. *Molecular Medicine Today*.

[16] Nath S, Daneshvar K, Roy LD (2013). MUC1 induces drug resistance in pancreatic cancer cells via upregulation of multidrug resistance genes. *Oncogenesis*.

[17] Mariani G, Molea N, Bacciardi D (1995). Initial tumor targeting, biodistribution, and pharmacokinetic evaluation of the monoclonal antibody PAM4 in patients with pancreatic cancer. *Cancer Research*.

[18] Gnoni A, Licchetta A, Scarpa A (2013). Carcinogenesis of pancreatic adenocarcinoma: precursor lesions. *International Journal of Molecular Sciences*.

[19] Zamboni G, Hirabayashi K, Castelli P (2013). Precancerous lesions of the pancreas. *Best Practice & Research Clinical Gastroenterology*.

[20] Gold DV, Goggins M, Modrak DE (2010). Detection of early-stage pancreatic adenocarcinoma. *Cancer Epidemiology Biomarkers and Prevention*.

[21] Gold DV, Modrak DE, Ying Z, Cardillo TM, Sharkey RM, Goldenberg DM (2006). New MUC1 serum immunoassay differentiates pancreatic cancer from pancreatitis. *Journal of Clinical Oncology*.

[22] Gold DV, Gaedcke J, Ghadimi BM (2013). PAM4 enzyme immunoassay alone and in combination with ca19-9 for the detection of pancreatic adenocarcinoma. *Cancer*.

[23] Gold DV, Alisauskas R, Sharkey RM (1995). Targeting of xenografted pancreatic cancer with a new monoclonal antibody, PAM4. *Cancer Research*.

[24] Gold DV, Cardillo T, Goldenberg DM, Sharkey RM (2001). Localization of pancreatic cancer with radiolabeled monoclonal antibody PAM4. *Critical Reviews in Oncology/Hematology*.

[25] Alisauskus R, Wong GYC, Gold DV (1995). Initial studies of monoclonal antibody PAM4 targeting to xenografted orthotopic pancreatic cancer. *Cancer Research*.

[26] Cardillo TM, Ying Z, Gold DV (2001). Therapeutic advantage of ^90^yttrium- versus 131iodine-labeled PAM4 antibody in experimental pancreatic cancer. *Clinical Cancer Research*.

[27] Gulec SA, Cohen SJ, Pennington KL (2011). Treatment of advanced pancreatic carcinoma with ^90^Y-clivatuzumab tetraxetan: a phase I single-dose escalation trial. *Clinical Cancer Research*.

[28] Chopra A (2011). ^111^In/^125/131^I-labeled anti-mucin-1 murine, chimeric or humanized antibody hPAM4. *Molecular Imaging & Contrast Agent Database*.

[29] Ocean AJ, Pennington KL, Guarino MJ (2012). Fractionated radioimmunotherapy with ^90^Y-clivatuzumab tetraxetan and low-dose gemcitabine is active in advanced pancreatic cancer: a phase 1 trial. *Cancer*.

[30] Cardillo TM, Karacay H, Goldenberg DM (2004). Improved targeting of pancreatic cancer: experimental studies of a new bispecific antibody, pretargeting enhancement system for immunoscintigraphy. *Clinical Cancer Research*.

[31] Chopra A (2011). ^125^I-labeled anti-mucin 1 bispecific antibody bsPAM4. *Molecular Imaging & Contrast Agent Database*.

[32] Chopra A (2011). ^99 m^Tc-Ac-Lys(DTPA)-Tyr-Lys(DTPA)-Lys (thiosemicarbazonyl-glyoxyl-cysteinyl-)-NH2 (IMP-192) [[^99 m^Tc]-IMP-192]. *Molecular Imaging & Contrast Agent Database*.

[33] Chopra A (2011). ^111^In-labeled Ac-Phe-Lys(DTPA)-Tyr-Lys(DTPA)-NH2 (IMP-156) [[^111^In]-IMP-156]. *Molecular Imaging & Contrast Agent Database*.

[34] Gold DV, Goldenberg DM, Karacay H (2008). A novel bispecific, trivalent antibody construct for targeting pancreatic carcinoma. *Cancer Research*.

[35] Chopra A (2011). ^111^In-labeled 1, 4, 7, 10-tetraazacyclododecane-N, N', N”, N”'-tetraacetic acid (DOTA)-D-Tyr-D-Lys(HSG)-D-Glu-D-Lys(HSG)-NH2 (IMP-288) [[^111^In]-IMP-288]. *Molecular Imaging & Contrast Agent Database*.

[36] Chopra A (2011). ^125^I-labeled trivalent, bispecific monoclonal antibody construct TF10 that targets mucin-1 and is reactive against a histamine-succinyl-glycine hapten IMP-288 [[^125^I]-TP10]. *Molecular Imaging & Contrast Agent Database*.

[37] Gold DV, Cardillo T, Vardi Y, Blumenthal R (1997). Radioimmunotherapy of experimental pancreatic cancer with 131I-labeled monoclonal antibody PAM4. *International Journal of Cancer*.

[38] Gold DV, Modrak DE, Schutsky K, Cardillo TM (2004). Combined ^90^Yttrium-Dota-Labeled PAM4 antibody radioimmunotherapy and gemcitabine radiosensitization for the treatment of a human pancreatic cancer xenograft. *International Journal of Cancer*.

[39] Cardillo TM, Blumenthal R, Ying Z, Gold DV (2002). Combined Gemcitabine and radioimmunotherapy for the treatment of pancreatic cancer. *International Journal of Cancer*.

[40] Gold DV, Schutsky K, Modrak D, Cardillo TM (2003). Low-dose radioimmunotherapy (^90^Y-PAM4) combined with gemcitabine for the treatment of experimental pancreatic cancer. *Clinical Cancer Research*.

[41] Karacay H, Sharkey RM, Gold DV (2009). Pretargeted radioimmunotherapy of pancreatic cancer xenografts: TF10- ^90^Y-IMP-288 alone and combined with gemcitabine. *Journal of Nuclear Medicine*.

[42] Glazer ES, Zhu C, Massey KL (2010). Noninvasive radiofrequency field destruction of pancreatic adenocarcinoma xenografts treated with targeted gold nanoparticles. *Clinical Cancer Research*.

